# Artificial intelligence-based tools for precision diagnosis and treatment of neurofibromatosis type 1 associated peripheral and central glial tumors

**DOI:** 10.1186/s13023-025-04093-5

**Published:** 2025-10-30

**Authors:** Fabio Hellmann, Inka Ristow, Lennart Well, Swanhild Lohse, Maxim Anokhin, Michaela Kuhlen, Elisabeth André, Anja Harder

**Affiliations:** 1 Human-Centered Artificial Intelligence, University of Augsburg, Universitaetsstrasse 6a, 86159 Augsburg, Bavaria Germany; 2 Department of Diagnostic and Interventional Radiology and Nuclear Medicine, University Medical Center Hamburg-Eppendorf, Martinistrasse 52, 20251 Hamburg, Germany; 3 Medical Faculty, Martin-Luther-University Halle-Wittenberg, Grosse Steinstrasse 52, 06108 Halle, Saxony-Anhalt Germany; 4 Pediatrics and Adolescent Medicine, Faculty of Medicine, University of Augsburg, Stenglingstr. 2, 86156 Augsburg, Bavaria Germany; 5 Cure NF Research Group, Medical Faculty, University of Münster, Albert-Schweitzer-Campus 1, 48149 Münster, North Rhine-Westphalia Germany

**Keywords:** Neurofibromatosis, Tumors, Artificial intelligence, Deep learning

## Abstract

Modern Artificial Intelligence (AI) has demonstrated its effectiveness by achieving human-level performance in various complex tasks, including the biomedical field. Cancer research, adapting to a fast-changing world, is leveraging AI as a promising framework to better understand tumor development. Moreover, current AI methods can help predict more suitable and personalized treatment strategies for specific types of tumors. We explored AI methods applied to Neurofibromatosis Type 1, focusing on glial tumors. Additionally, we have reviewed all publicly available datasets to date. Discussion of future challenges is highly desirable since Neurofibromatosis Type 1 is one of the most common hereditary tumor syndromes and is associated with an increased rate of glial tumors as well as a reduced life expectancy due to malignancy.

## Background and rationale

In recent years, clinical and research interest in Artificial Intelligence (AI) has increased dramatically. This has also had an impact on Neurofibromatosis (NF). NF comprises the genetic disorders Neurofibromatosis Type 1 (NF1) and Schwannomatosis (SWN). SWN is classified according to the specifically involved gene harboring a pathogenic variant, such as *NF2*-related SWN, *SMARCB1*-related SWN, *LZTR1*-related SWN, 22q-related SWN, and SWN-NOS (not otherwise specified) or SWN-NEC (not elsewhere classified). They all share the development of glial tumors of the Central Nervous System (CNS) and/or Peripheral Nervous System (PNS). Autosomal dominant inherited NF1 is the most prevalent of these neurocutaneous disorders and is associated with manifestations in the skin, ophthalmic, musculoskeletal, nervous system, bone, vascular, and other systems. Those patients are prone to develop neurofibromas, dominating the disease. Neurofibromas mainly consist of Schwann cells and fibroblasts, and the Schwann cell represents the peripheral counterpart of the CNS glia. It is important to note that NF1 patients have an increased risk of about 20% of CNS tumors, particularly glial tumors [1–3].

Diagnosing NF1-associated tumors can be challenging due to their diverse clinical manifestations and unpredictable growth patterns. Conventional diagnostic tools, such as biopsy, are invasive. Using imaging, a definitive diagnosis may not be possible. AI-based tools, particularly those employing Machine Learning (ML) and Deep Learning (DL) algorithms, have shown promising results in both detecting and diagnosing various tumors. These tools can analyze large volumes of data, identify subtle patterns that may be overlooked by human observers, and provide real-time predictions.

Still, most research relies on more comprehensible and transparent ML algorithms such as decision trees, random forest regression, or similar [4–6]. The literature is very sparse, considering the previous research on NF1 and DL. Ho et al. showed that DL was successfully applied in a semi-automated process with a neural network to generate volume maps based on 35 Magnetic Resonance Imaging (MRI) scans [7]. Furthermore, the team of Artzi et al. combined ML and DL methods for automatic segmentation and classification of chiasmatic Optic Pathway Gliomas (OPG) based on 202 conventional MRI scans from 29 patients [8]. Another approach to an interactive DL model for segmenting neurofibroma on Whole-Body (WB) MRI was proposed by Zhang et al., which was solely based on 3 MRI scans [9]. The amount of data available in the majority of DL studies is very limited, which hinders research on NF1 using DL. However, this limitation can be overcome through more intense collaborations with clinical partners, as it is imperative to utilize DL very early in diagnosing NF1 and its associated manifestations.

**Fig. 1:**
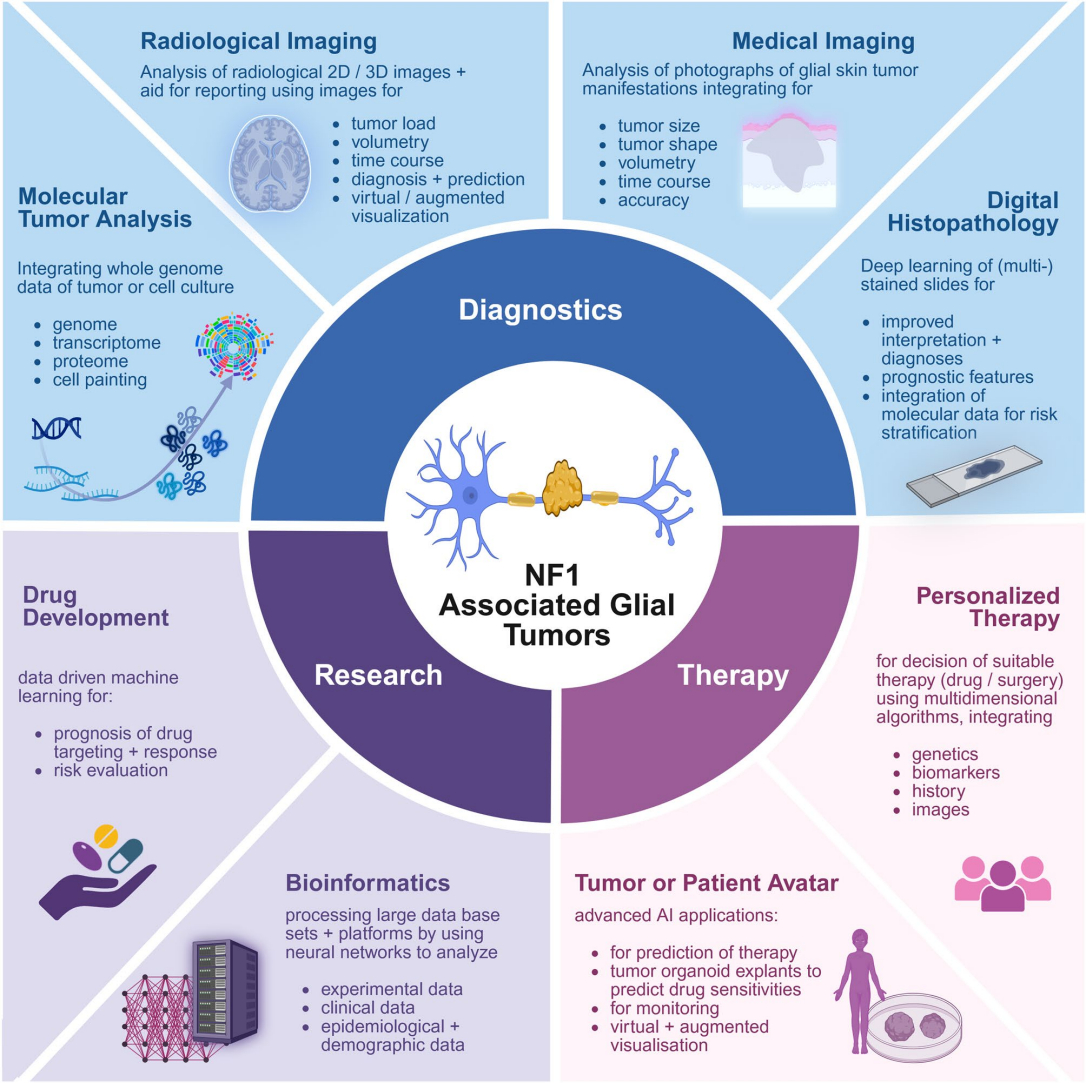
Potential applications of AI in NF1 concerning glial tumors - current state and challenges. Created in BioRender. Lohse, S. (2025) https://BioRender.com/g8ep4zo (SI28VREM38, 16.10.2025)

**Fig. 2:**
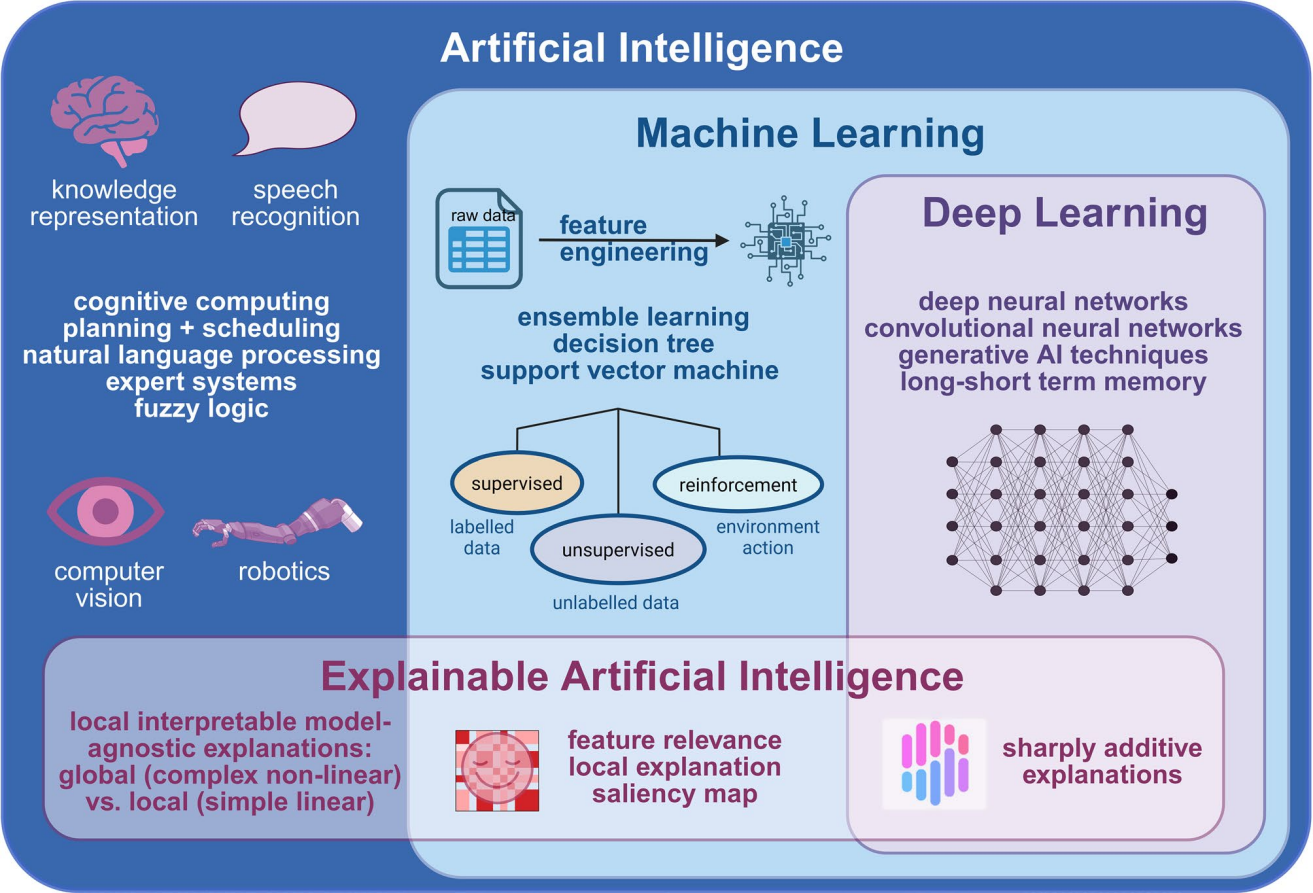
Subfields and applications of artificial intelligence. Created in BioRender. Lohse, S. (2025) https://BioRender.com/45gpcix (UO288KVOQA, 7.5.2025)

Methods that utilize AI support already play a role in many fields associated with NF1, similar to other oncologic, neurocutaneous, or chronic diseases (Fig. 1). This might include large language models, assistants for writing, presenting, analyzing, and designing medical and scientific work, decision-augmenting software to improve diagnostics (e.g., MoleAnalyzer Pro for dermatoscopy  [10], Face2Gene  [11] or GestaltMatcher [12] for phenotypes, NFsimplified  [13] to translate scientific literature into patient-friendly summaries, or enhancing decision-making in molecular precision therapy). More than 500 AI-based medical applications are currently listed by the US Food and Drug Administration (FDA). However, this does not accurately reflect the current status of medical applications in Europe. In this paper, we focus on applications explicitly associated with new developments in the field of NF1, particularly peripheral glial (Schwann cell-derived) and central glial tumors (astrocytomas), which are typically linked to the disease. We look at two major fields that are rapidly developing: AI-based pathological diagnostics and related decisions, and imaging of tumors associated with NF1. We briefly introduce the terminology of the AI field and NF1-associated glial tumors (Fig. 2). Afterwards, we focus on diagnostics of NF1-associated glial tumor manifestations, challenges, datasets, and review the current state of NF1 in Imaging to propose future directions with AI.

### Background on artificial intelligence

Artificial Intelligence is an essential tool in precision medicine, and its subfields - Machine Learning, Deep Learning, and Explainable Artificial Intelligence - are displayed in Fig. 2. AI refers to the development of computer systems that can perform tasks typically requiring human intelligence, such as perception, reasoning, learning, and decision-making.

#### Machine learning

Machine Learning is a subset of AI that involves the development of algorithms that can learn from data and improve their performance over time without being explicitly programmed. ML algorithms can identify patterns in data and make predictions based on those patterns. However, before it is possible to identify those patterns accurately, it is crucial to engineer the features for training the ML algorithms. Feature engineering is the process - executed by a person or group of persons - of selecting, transforming, and extracting relevant features from raw data to improve the performance of ML models. It involves identifying and creating new informative, discriminative, and useful features for the learning task. Feature engineering aims to represent the data in a way that captures the underlying patterns and relationships, enabling the model to make accurate predictions or classifications. Feature engineering often requires domain expertise, creativity, and experimentation to achieve optimal results. After manually engineering the features, the ML algorithm learns in a supervised or unsupervised way. Supervised learning algorithms learn from labeled data by identifying the optimal output for each input. The algorithm uses this labeled data to learn a function that can map new inputs to the correct output. On the other hand, unsupervised learning algorithms do not rely on labeled data. Instead, they identify patterns and structures in the data to uncover hidden relationships or groupings.

Generally, these types of models can be utilized for classification and segmentation tasks. Traditional segmentation techniques involve thresholding, region growing, and edge-based methods. These methods use simple intensity or gradient-based rules to partition images into distinct areas. ML-based methods can also be used for segmentation tasks, including decision trees, random forests, and support vector machines. These techniques leverage handcrafted features and trained models to classify pixels or voxels into different classes.

#### Deep learning

Deep Learning is a subset of ML that involves the development of neural networks, which are computational models inspired by the structure and function of the human brain. Deep Neural Networks consist of multiple layers, each processing the input from the previous layer. The first layer processes raw input data, such as an image or audio file or manually engineered features, and subsequent layers extract increasingly complex features from the input. The output of the final layer is the prediction made by the network. The feature engineering step is embedded into the DL algorithm itself. Accordingly, the DL algorithm extracts the relevant features from the raw data that seem fit to match the provided output labels. DL has proven to be particularly effective in tasks that require recognizing complex patterns, such as image and speech recognition.

Like in ML, DL can also be used for segmentation by utilizing architectures like U-Net  [14], Fully Convolutional Networks  [15], and DeepLab  [16]. These networks can learn complex spatial patterns and have demonstrated remarkable success across various medical imaging tasks. While many segmentation methods focus on 2D images [8], medical applications often require segmenting volumetric data in three dimensions. Techniques such as 3D convolutional networks and volumetric extensions of 2D architectures address the challenges of 3D segmentation [7, 9]. DL approaches offer increased accuracy, reduced human intervention, and the potential for real-time applications.

Volumetric analysis and 3D imaging enable a comprehensive understanding of complex anatomical structures and pathologies. Techniques like 3D convolutional networks and region-based methods extend traditional 2D segmentation approaches to volumetric data, accounting for spatial context and continuity. Networks like 3D U-Net  [17] and V-Net  [18] leverage volumetric information to enable accurate segmentation, detection, and classification tasks. An emerging trend indicates that volumetric analysis involves integrating AI with virtual and augmented reality for immersive visualization.

#### Explainable artificial intelligence

Explainable Artificial Intelligence (XAI) is a field that seeks to make AI algorithms more transparent and understandable to humans by providing insights into how AI systems arrive at decisions. XAI is particularly important in fields such as healthcare, where it is essential to understand the reasoning behind an AI system’s diagnosis or treatment recommendation [19]. One approach to XAI is to develop inherently interpretable models, such as decision trees or linear regression models [20]. These models are relatively simple and easy to interpret, allowing for a clear understanding of how they arrive at their decisions. Another approach to XAI is to develop post-hoc explanations for black-box models, such as DL networks [20]. These explanations seek to provide insights into why a particular decision was made by examining the weights and activations of the network. For example, in image classification, an XAI technique could highlight the image’s most influential parts in the network’s decision. An up-and-coming line of work in medical XAI involves the generation of counterfactual explanations, which aim to explain which minimal changes in the input would have led to a different diagnosis [21]. Such approaches can help both clinicians and patients better understand the model’s behavior and its decision boundaries.

### Glial tumors in neurofibromatosis type 1

Neurofibromas comprise both haploinsufficient (*NF1* +/-) and neoplastic (*NF1* -/-) Schwann cells, accompanied by immune and perineurial-like cells, neurons, various types of vessel cells, fibroblasts and collagen. They arise during Schwann cell maturation depending on intrinsic and extrinsic factors [22]. Neurofibromas comprise plexiform, localized cutaneous, diffuse cutaneous, localized intraneural (subcutaneous), and other subtypes of tumors [23]. The localized cutaneous neurofibroma represents a well-known, benign key feature. The Plexiform Neurofibroma (PNF) exhibits complex biological behavior and is prone to atypical and malignant transformation. Although primarily benign, PNF can cause considerable pain, disfigurement, and life-threatening complications. Standard surgical treatment is now being accompanied by new targeted therapies, such as with MEK1/2 (Mitogen-activated protein kinase kinase 1/2) inhibitors (MEKi) and multireceptor tyrosine kinase inhibitors [24–28]. In 2020, selumetinib became the first medication approved by the US FDA to treat pediatric patients with symptomatic, inoperable PNF associated with NF1. Recently, mirdametinib was approved to treat adult patients with incompletely resected PNFs [29]. Identifying a change towards atypia in NF1-associated peripheral glial tumors is challenging [23]. Those atypical neurofibromas (or Atypical Neurofibromatous Neoplasm with Uncertain Biological Potential, ANNUBP) might already accumulate *CDKN2A/B* alterations. Still, it might demonstrate only subtle histological differences or borderline imaging features, or might not be biopsied adequately. Here, AI-based technologies could be beneficial in making diagnostics much safer and independent of the observer.

Apart from neurofibromas, the most frequent neoplasms in children with NF1 are low-grade gliomas (mainly pilocytic astrocytomas), typically located in the optic pathway and brainstem [30]. Although these tumors often display a benign clinical course, treatment is necessary for patients with a threat to vision and progressive disease. The standard of treatment for low-grade glioma is surgical resection. However, surgical resection is not an option in many cases due to the predominant localization of the optic pathway and brainstem in NF1 patients. Conventional therapeutic approaches comprise vincristine and carboplatinum-based chemotherapy regimens. In a phase II study in 25 pediatric NF1 patients with recurrent, refractory, or progressive low-grade glioma, 40% of the patients had $\geq 50\%$ tumor reduction with selumetinib, and only one patient had progressive disease [31].

Malignant glial tumors pose a particular challenge. Malignant Peripheral Nerve Sheath Tumors (MPNST) are highly aggressive sarcomas that represent the most frequent malignancy in patients with NF1 and the leading cause of mortality. MPNSTs are refractory to conventional chemotherapy and radiation. Here, too, surgical resection is the mainstay of therapy. However, the outcome is dismal in incompletely resected MPNSTs or cases of metastases. Novel treatment approaches, such as everolimus and bevacizumab, or sirolimus and ganetespib, exploring molecular pathways implicated by mutations in NF1, have not yet yielded any additional benefit [32, 33]. Results of a study evaluating the combination of mTOR and MEK inhibition by using selumetinib and sirolimus are pending (NCT03433183).

Malignant CNS tumors are discussed in more detail in the next chapter. Nevertheless, they generally show a worse prognosis in NF1 compared to non-NF1 patients.

As in peripheral tumors, complete loss of function of the *NF1* gene product, neurofibromin, is mandatory for CNS tumor initialization. Still, other genetic aberrations such as *TP53* loss, *ATRX* mutations, *CDKN2A/B* homozygous loss or mutation, *PIK3CA* or *PIK3R1* mutation, *SUZ12* deletion, as well as the *NF1* haploinsufficient environment and immune system, are drivers for tumor development. NF1-associated CNS gliomas, therefore, typically arise from consequences of RAS/MAPK pathway alterations and show the absence of alterations of other genes that are associated with glial tumor development outside of NF1, such as *BRAF, TERT, EGFR, IDH,* and histone H3 mutations [34]. Glial CNS tumors arising in NF1 are specified in Table 1 concerning the current World Health Organization (WHO) classification system [35]. Beyond this professionally binding textbook, there are some new reports addressing other CNS tumor types not covered so far concerning NF1, such as embryonal tumors [36, 37], e.g., embryonal tumor with multilayered rosettes, diffuse low-grade astrocytoma (WHO grade 2–3), SEGA-like astrocytomas, diffuse midline glioma H3K27M mutant, and low-grade astrocytoma with interdeterminate subtype [1, 37]. Lucas et al. [34] suggested two subgroups arising from molecular testing: indolent / primarily childhood gliomas with biallelic *NF1* inactivation of the *NF1* gene (epigenetically “pilocytic astrocytoma, arising in the setting of NF1”), and aggressive / primarily adult gliomas with further genetic alterations such as *CDKN2A* homozygous deletion or *ATRX* mutation (epigenetically mostly high-grade astrocytoma with piloid features or subclasses of IDH-wildtype glioblastoma). Other studies have shown that a percentage of childhood low-grade gliomas exhibit further alterations, such as those involving *FGFR1* [38]. Among NF1-associated gliomas, pilocytic astrocytomas of the optic pathway are the most common and primarily affect young children, necessitating a thorough diagnostic workup. Glioblastomas that harbor *NF1* variants show a specifically poor outcome [39].

**Table 1:** Glial central nervous system tumors in neurofibromatosis type 1 according to the current world health organization (WHO) classification [35]

**Tumor type / subtype** (WHO grading, if reported)	Aberrations of genes involved other than biallelic NF1 loss
**Circumscribed astrocytoma**	
Pilocytic astrocytoma (grade 1)	
High grade astrocytoma with piloid features	*ATRX*
Pilomyxoid astrocytoma	
**Pediatric-type diffuse low-grade astrocytoma**	
Diffuse low-grade glioma, MAPK pathway-altered	
**Pediatric-type diffuse high-grade astrocytoma**	
Glioblastoma, IDH wild-type (grade 4)	*TP53*, *CDKN2A/B*, *ATRX*
**Glioneuronal tumor (different types)**	

Since NF1-associated optic gliomas exhibit specific growth dynamics during childhood, decisions regarding diagnostics and treatment are challenging. Gliomas other than pilocytic astrocytomas, also associated with NF1, display a worse prognosis. Therefore, specific solutions for this group of patients are also required and differ from those for other cancer patients. Reevaluating existing datasets and utilizing AI for secure histological diagnosis may enhance subtype identification and the specification of therapies for NF1.

## Review methodology

This narrative review integrates clinical and technical dimensions of AI applications in NF1-associated glial tumors. The methodology was designed to address the heterogeneity of AI approaches across pathology, imaging, and molecular diagnostics while maintaining reproducibility.

### Search strategy

A comprehensive search strategy was deployed across the databases: PubMed/MEDLINE and Google Scholar. The search period spanned from the database’s inception through February 2025, with the final query executed on June 27, 2025. Keywords focused on the intersection of NF1, glial tumors, and AI techniques, e.g., specifically:


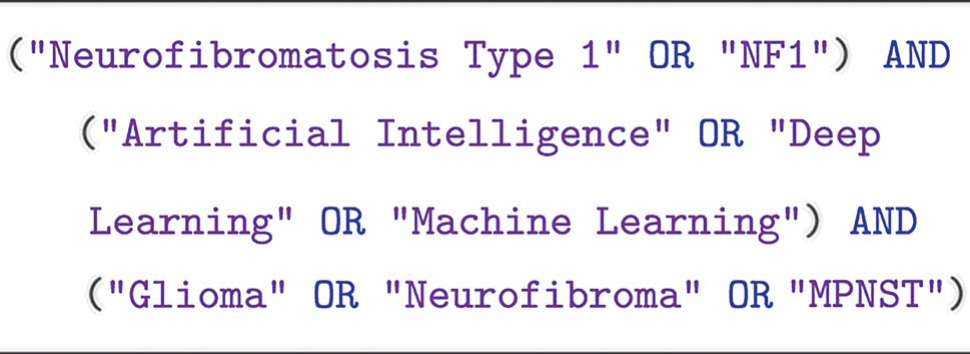


Filters restricted results to human studies, English-language publications, and peer-reviewed articles/conference abstracts.

### Inclusion/exclusion criteria

Inclusion criteria only considered primary research, clinical trials, and reviews addressing AI applications in NF1-associated peripheral/central glial tumors. Exclusion criteria removed animal studies, non-computational methods, and purely biological investigations. The study selection process included a two- to three-fold independent screening, as well as manual additions from SYNAPSE, the CTF data portal, and BioArchives, to collect technical reports and preprints.

### Quality assessment

Technical studies underwent rigorous quality assessment using reproducibility metrics (code availability, hyperparameter documentation).

## AI applications in pathology

### Conventional pathology

Histopathological images provide detailed information about the cellular composition and architecture of tumors, which is crucial for diagnosis and treatment planning. However, manual analysis of these images can be time-consuming and subjective. AI algorithms, particularly those based on DL, can learn to recognize intricate patterns and features within histopathological images, enabling precise tumor classification and grading [40]. These DL models are trained on diverse datasets of histological images, allowing them to differentiate between various tumor types and grades based on subtle visual cues that human observers may not easily discern.

**Fig. 3:**
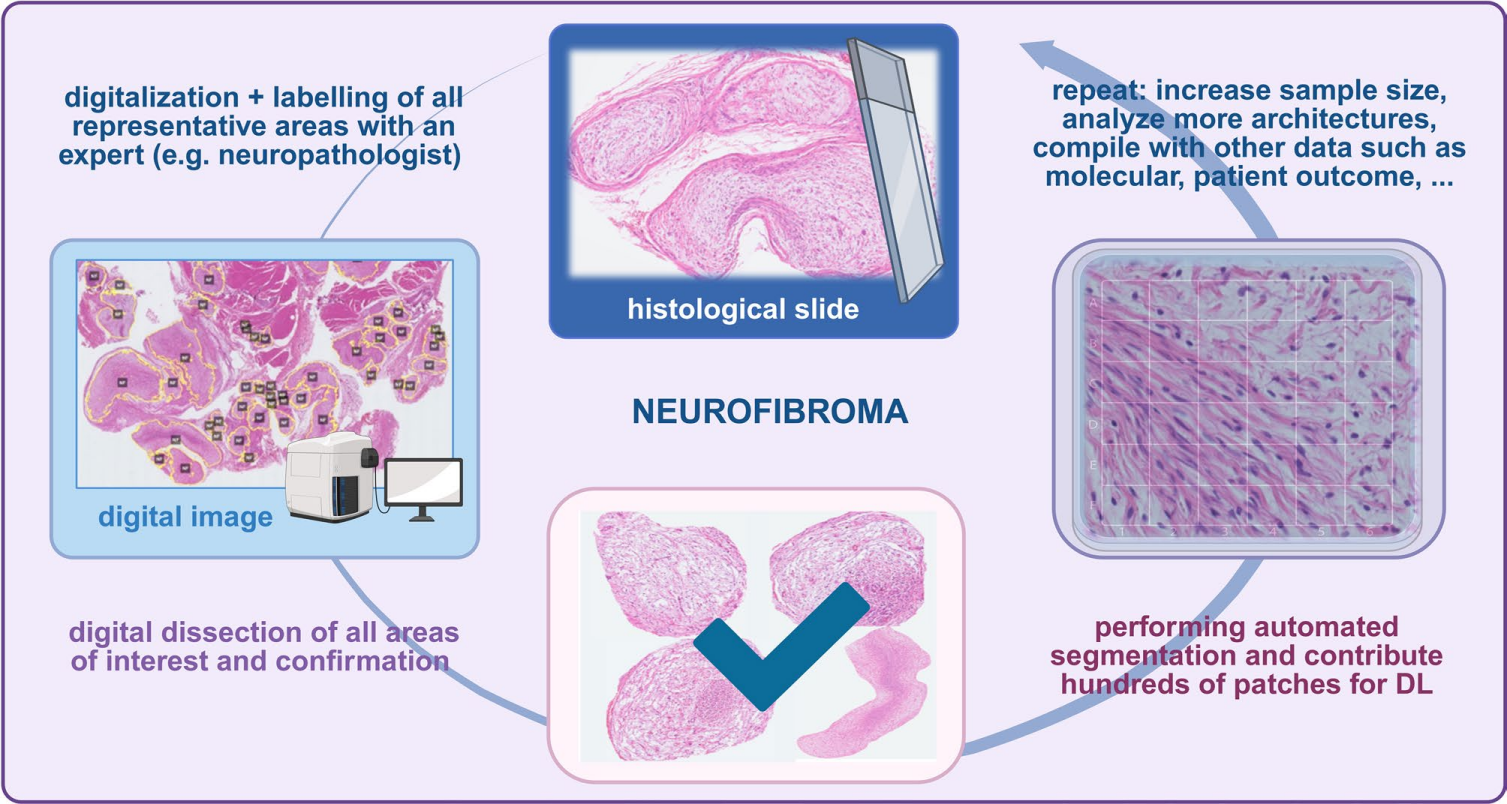
General procedure on how digital whole slide images (WSI) of peripheral nerve sheath tumors can be subjected to AI training using deep learning algorithms. Created in BioRender. Lohse, S. (2025) https://BioRender.com/e83n768 (YE288KWFMN, 7.5.2025)

The histopathological diagnosis of tissue from NF1 patients, such as nerve sheath tumors, brain tumors, or other lesions, remains primarily based on hematoxylin and eosin-stained Formalin-Fixed Embedded Tissue (FFPE) sections, immunohistochemistry, and, increasingly, molecular tissue-based diagnostics. Specimens are mainly analyzed with a microscope or digitized Whole-Slide Image (WSI) images. This work is subjective, labor-intensive, and time-consuming, and depends heavily on the neuropathologist’s technical and clinical experience, training, and workflow. Subjectivity and reproducibility of WSI interpretation can potentially be overcome by DL processing tasks and extracting a complex hierarchy of features. In this field, tumor segmentation and classification methods in WSI using DL have developed significantly in recent years; however, they have been applied to NF1-associated tumors only rarely. But, DL-based approaches have become powerful and have already been developed in several other diseases and model organisms. In a mouse model, an automated DL algorithm recognized subtle differences in the liver parenchyma, excluded vessels and artifacts, and quantified areas in WSIs of HE sections with high reproducibility, enabling the detection of forms of liver steatosis. In principle, histological slides are easy to scan and segment (Fig. 3). However, the fine-grained segmentation, done by physicians, can be very time-consuming and complex. Therefore, DL algorithms could contribute to the automated diagnosis, still leaving the responsibility to the pathologist in complicated cases.

Besides NF1-associated diagnostics in glial tumors, several approaches are already using AI-based analyses for histological slides: attention-based multiple-instance learning approach (attMIL) to predict molecular alterations and subtypes of brain tumors from histopathology images [41], attention-based Convolutional Neural Network (CNN)s to classify meningiomas from histopathological images [42], deep neural networks / domain-specific ResNet architectures for classifying spinal cord ependymomas [43], DL and ML for image processing in peripheral nerve histomorphometry [44], and automated segmentation of myelinated peripheral nerve fibers using a AxonDeepSeg framework, and many other studies in the field of pathology. However, few publications concern histological analyses of NF1-associated tumors and tissues. Oldridge and coworkers presented a machine-learning approach to identify tumor-infiltrating lymphocytes in NF1-associated gliomas, MPNST, and various types of neurofibromas, which had not been previously published (personal communication at poster presentation, CTF congress 2022). A morphometric analysis using an open source software QuPath [45] for digital pathology image analysis of glioblastomas with the selection of morphometric cell clusters detected four morphometric clusters differing in morphometric, functional, and molecular parameters as well as a separate subcluster of stem cell elements corresponding to stem cells in all four clusters. In this study, altered NF1 gene cell clusters were identified. QuPath is a framework for the training and application of advanced AI on a high level, as shown in work identifying classifiers of tumor- and non-tumor cells in lung cancer [46].

### Tissue-based analyses, datasets, and molecular pathology

In a recent study, Raman spectroscopy, a molecular spectroscopic technique, was applied to specimens of cutaneous neurofibromas and other tissues to identify the border of neurofibroma from other physiological entities and optimize surgical procedures. It was possible to distinguish the tumor from other tissue using the isolation SERDS technique in conjunction with a U-Net CNN [47]. Further tissue-based studies have the potential to provide detailed information about the chemical and molecular composition of biological tissue in the future. Hopefully, multistain DL will be able to address not only a precise histological diagnosis but also prognostic capabilities by using a set of several additional information, such as genetic germline information, molecular background, specific features such as immune cell infiltration, and so on, as it was shown for colon cancer recently [48].

Using extracted data from the Cancer Genome Atlas (TCGA) PanCancerAtlas and ML, Way and co-authors were able to detect MEKi sensitivity and Ras Pathway activation, which is also typical of NF1-associated tissue [49]. The same group recently demonstrated new options for predicting NF1 genotypes in cell culture. They used high-content microscopy and cell painting of organelles of cultured Schwann cells for feature extraction and ML to predict an NF1 genotype, e.g., targeted drug testing [50]. A DL model has been trained on subtypes of glioblastoma that might be of prognostic relevance to represent biologically and clinically low-dimensional representations of cancer gene expression data, which might also be of interest for NF1-associated glioblastoma patients [51]. Another AI model was established to predict benign and malignant tumors in NF1-associated PNS tumors using genomics, imaging, and demographic data by DL and a dataset from a study that performed analyses of neurofibromatosis-associated tumors and identified SOX9 as a biomarker [52, 53]. A machine-learning approach of cell-free DNA features was also used to classify malignant and premalignant cells from nerve sheath tumors [54]. The method identified fragmentomic signatures to distinguish atypical neurofibromas from benign PNFs, and MPNST is powerful for precision management in NF oncology patients. The development of the Healx Healnet platform to identify novel treatments in NF1 is a promising approach that utilizes AI directly in NF1 for drug discovery and application [55]. Although there is currently no evidence of genuine clinical added value or validated effectiveness of the application, such evidence appears likely in the near future due to promising approaches (phase II studies for selected candidates and approval as an investigational new drug by the US FDA in August 2024). Additionally, transcriptomic data of 77 NF1-associated tumors retrieved from the NF Data Portal were also used in the context of latent variables from MultiPLIER, which serves as an ML tool to identify candidates for drug development, gene expression variables, as well as immune and protein activity signatures [56]. Drug response prediction driven by AI is an emerging field, and multi-omics can serve as an indispensable tool used for DL algorithms. However, some experienced scientists criticize the data sets as being poorly prepared and in need of harmonization [57]. After all, the first steps nicely demonstrate how harmonization can accelerate data exploration in NF1 [58, 59].

Genomic data from NF1 patients and NF1-associated tumors can be segmented to detect similarities between otherwise distinct patients and their samples, or differences between similar samples. This approach is desirable as molecular data segmentation will help identify targeted therapies. To achieve this, NF-specific data-sharing platforms have emerged, such as SYNAPSE  [60], which provide information and facilitate collaboration and the use of specific NF-related datasets. More than 60 results are related to NF1, and over 170 are related to brain tumors. Although not being used for AI-based applications, projects described on SYNAPSE, such as “factors that define the pattern of glioma formation in NF1”  [60] or the Children’s Tumor Foundation (CTF) data portal, might be significant for further evaluation.

For example, the Open Pediatric Brain Tumor Atlas [61] was utilized by an open science initiative to define the molecular landscape of 943 tumors. Participants were derived from the Children’s Brain Tumor Network [62] and the clinical trial of the Pediatric Pacific Neuro-Oncology Consortium [63]. RNA-seq data were used in combination with histology and diagnostic information, and Graph Neural Networks, Deep Walk, Metapath2vec, and t-distributed stochastic neighbor embedding (t-SNE) were applied to build a classification model and improve sample/gene/patient representations [64]. One limitation of this approach seems to be the heterogeneous quality of the data from OpenPBTA, which is not handled by representation learning models such as metapath2vec [65]. Other studies using AI methodology focused on decision-making for everybody in health care in the context of rare diseases to avoid umbrella diagnosis and treatment: QuDoc was developed to allow precision-based diagnosis of a rare disease using quantum ML, although so far clinical validation or patient-relevant outcomes appear not to be available [66]. Data on risk factors, symptoms, birth, gender, and much more are assessed via a mobile interface that finally integrates data and produces a reliable probability of a rare disease.

As molecular data comprises genetics and molecular profiling information, they provide essential insights into how diseases develop and how patients respond to treatment. AI algorithms can analyze large sets of molecular data and have the potential to discover patterns that could indicate outcomes for NF1 patients, the effectiveness of their therapies, or the risk of certain other complications in NF1. Already in 2014, ML was tested to predict clinical NF1 features that are important for risk stratification of NF1 patients [4]. Regarding NF1, AI models can learn from various samples and complex molecular interactions, enabling them to identify subtle cues that traditional analysis methods may miss.

## AI applications in imaging

In this section, we will provide an insight into the clinical imaging of today’s physicians. Furthermore, we will highlight the challenges physicians face with today’s imaging techniques and provide insight into how AI can help overcome those difficulties.

### Non-radiological AI-based imaging analysis

As mentioned, cutaneous neurofibroma is the hallmark manifestation of NF1. The clinical heterogeneity is high. As novel treatments emerged, it seems vital to precisely follow tumor manifestation and growth, e.g., by evaluating images from patients’ skin with neurofibromas. This is currently being investigated in an international collaboration among Johns Hopkins, Queensland, and Sydney universities, utilizing ML and DL for segmentation [67], which evaluates 2D photography of NF1 patients.

### Radiological imaging - overview

NF1 patients are potentially subjected to various imaging studies throughout their lives due to the diverse manifestations of their condition.

Cutaneous neurofibromas pose enormous cosmetic and quality-of-life issues and are, therefore, a target for new therapeutic developments alongside conventional surgical methods. AI-based analysis of 2D and 3D images of neurofibroma manifestation on the WB can be a powerful tool for achieving reliable results in new treatments.

Typical skeletal changes, such as scoliosis and deformities of long bones, are examined using conventional X-ray. MRI is crucial in investigating tumors associated with disease. It is the preferred imaging modality in NF1 patients as they are at increased risk for developing secondary tumors after radiation exposure. 18F-fluorodeoxyglucose (18FDG) Positron Emission Tomography (PET), MRI, and 18FDG PET Computed Tomography (CT) are essential for addressing questions related to tumor malignancy. The clinically most relevant tumors in NF1 are PNF and OPG. Standard 1- or two-dimensional tumor growth criteria have limited value in detecting small volume changes in PNF due to their often enormous size and complex shape compared to other solid tumors. Similarly, growth assessment of OPG by conventional uni- or two-dimensional measurement is of limited value. Additionally, the diagnostic reliability regarding the malignant transformation of PNFs to MPNST remains limited, despite the use of morphological and quantitative markers (e.g., Diffusion-Weighted Magnetic Resonance Imaging (DWI) and PET/CT). Therefore, AI/ML-based techniques have the potential to enhance diagnostic accuracy in NF1. In this regard, automated tumor segmentation, volumetry, and radiomics-based approaches are particularly interesting.

AI-driven radiomics is built on its capability to extract various numerical details from medical images, like texture, shape, and intensity changes. These details reveal complex tumor patterns, highlighting their differences, blood supply, and surrounding environment. Traditional methods of studying these details manually are time-consuming, can vary between observers, and may not utilize all the valuable information in the images. CNNs have proven their ability to learn and extract complex patterns from these details [68]. These AI models are trained using large datasets from various types of tumors, enabling them to identify subtle patterns associated with specific tumor characteristics. With this knowledge, the models can later anticipate delicate characteristics such as tumor growth, blood vessel formation, tissue death, and invasion. AI-driven radiomics has an enormous potential in estimating how tumors react to treatments. By studying details from images taken before treatment, these models can provide insights into the chances of responding well to different treatments. This helps in picking the proper treatment and personalizing it. AI models’ forecasting power comes from their ability to capture detailed changes over time as treatment effects manifest as slight changes in tumor shape and texture. AI radiomics can detect these changes early on, helping to inform treatment strategies in a timely manner.

### Imaging-based diagnosis of plexiform neurofibromas and optic pathway gliomas

Plexiform Neurofibroma can be sharply delineated as hyperintense lesions from the surrounding tissue on a T2-weighted sequence with fat suppression. The presence of PNF and a high internal tumor burden are risk factors for developing an MPNST. Therefore, WB-MRI is the recommended imaging modality for long-term monitoring of PNF, and WB-MRI should be performed at least at the transition age to adulthood according to the current European Reference Network (ERN) non-Optic Pathway Gliomas (GENTURIS) tumor surveillance guidelines [69]. There are no specific protocol recommendations for WB-MRI examinations in the current ERN GENTURIS guidelines. However, in work by Ahlawat et al., the acquisition of coronal T1-weighted, coronal fat-saturated T2-weighted, and coronal or axial diffusion-weighted imaging sequences is recommended for WB-MRI [70].

In 2019, Uthoff et al. provided evidence that quantitative radiomics features are informative of the PNF malignancy status [71]. They identified T1 and T2 MRI intensity and texture features with significantly different feature expressions for plexiform benign and MPNST comparison. CT features were not found to be significant. Volumes of interest for feature evaluation were defined based on PET standard uptake value thresholds. Through cluster analysis, Liu et al. further illustrated that NF1 mutation types correlate with associated quantitative imaging features extracted from WB short tau inversion recovery MRI data [72]. Thus, a genotype-phenotype correlation exists. On the other hand, no correlation between tumor size and tumor location was found.

OPG are the most frequent CNS tumors in children with NF1, with an incidence of 15–20% [73]. MRI can depict OPG as an enlargement of the optic nerves, optic tract, or optic chiasm, with a relatively hyperintense signal on T2-weighted sequences. The current ERN GENTURIS guidelines recommend cranial MRI in patients whose ophthalmological examinations suggest OPG or are inconclusive. Additionally, cranial MRI is recommended for patients with clinical concerns of brain tumors to identify GENTURIS. Similar to WB-MRI, there are no specific protocol recommendations for cranial MRI in patients with NF1. However, the brain imaging guidelines of the European Society for Paediatric Oncology [74] and the Response Assessment in Paediatric Neuro-Oncology guidelines for imaging low-grade tumors [75] can serve as guidance. Automated segmentation models have also been developed for OPG. Compared to other CNS tumors (e.g., glioblastomas), the current segmentation performance is significantly lower, partly due to the often smaller datasets. Published OPG segmentation approaches achieved a DICE coefficient of $0.694 \pm 0.088$ in a study by Weizman et al., including 25 MRI datasets [76], $0.761 \pm 0.011$ in a study by Artzi et al. 2020 using 202 MRI datasets [8], and $0.799 \pm 0.047$ in a study by Nalepa et al. based on 494 MRI datasets [77]. Recently, an AI-powered web tool for MR-T1 volumetric analysis of NF1 OPG, called CAVS-NF1, was developed for free and easy use, based on SwinUNETR segmentation  [78]. This application is widely used in clinical studies (NF1-OPG natural history study, ACNS 1831). It has proven to be very fast (only 100 seconds), with a low volume error (approximately 16.7%), and more accurate than human performance.

### NF1 tumor load

MRI-based 3D volumetry has been established as a more powerful approach to assess internal tumor load in NF1 patients, but is associated with a significant time effort. Only a few automatic or semi-automatic methods have been proposed to speed up WB-MRI tumor segmentation. This may be explained by the challenges associated with rare diseases in general (i.e., small datasets and heterogeneous data due to long study acquisition periods) and NF1 itself, i.e., the manifold and often unique PNF manifestations in a substantial scan volume. In most of the approaches presented in the literature, interaction with the user was demanded to the extent that, for example, the area in which a tumor was to be segmented had to be predefined, an annotation of the center of each tumor had to be made, or, in retrospect, misclassified structures had to be excluded from the tumor volume [79]. The most recent work by Zhang et al. proposed a deep interactive network (3D U-Net) based on a training set of 125 WB-MRI studies and a testing set of 33 studies. However, fully automated PNF segmentation does not appear to be feasible at present. For example, Zhang et al. reported a volume overlap (measured by the Dice coefficient) of only 25% for 3D U-Net-based automated vs. expert segmentation [9]. Imaging studies have been primarily limited to segmentation, i.e., improved determination of tumor volume and tumor classification (benign vs. malignant). Further studies are needed, for example, to investigate the predictive value of imaging features on tumor growth or malignant transformation. However, this approach requires the analysis of feature variation at different acquisition time points (delta radiomics).

NF1-related AI-based studies are summarized in Table 2.

**Table 2:** Overview of AI techniques, maturity, robustness, metrics, and limitations in selected studies

Ref.	AI Techniques	Areas of AI	Methodological Robustness/Reproducibility	Metrics	Limitations
[8]	- U-Net + ResNet for segmentation - fuzzy c-means for components	- Deep learning & clustering	- Trained on 202 MRIs from 29 patients - 5-Fold CV - code not public	- Dice ≈0.76 - Precision ≈0.79 - Recall ≈0.74	- Single-center retrospective data - heterogeneous MRI parameters - distortion near skull base
[72]	- Random forest classifier	- AI in radiogenomics	- 29 patients (WB MRI & *NF1* gene mutation and tumor numbers) - Volumetric analysis - Statistical tests − 10-Fold CV - Data available on request	- 98.1% accuracy - 98.3% sensitivity - 98.0% specificity	- Small sample size - Imbalance in mutations
[76]	- Probabilistic tumor model - Spectral Angle Mapper (SAM), Generalized Likelihood Ratio Test (GLRT) used - Not Deep Learning	- Automatic OPG segmentation	- Quantitative evaluation (7 patients) - Training dataset used - Expert manual segmentations	- Volumentric overlap error 30.6% - Average symmetric surface distance 0.73 mm	- Small dataset - No external validation - Manual segmentation bias
[77]	- Deep Learning (nnU-Net, U-Net) - Pre-training on BraTS LGG/HGG MRI data - Transfer learning, data augmentation, ensemble averaging	- Automated segmentation	- 22 and 51 multi-modal MRIs with OPG +494 multi-modal MRIs with low-/high-grade glioma patients - Training details (e.g., optimizer, epochs, batch size) documented - CV on WAW (22 MRIs) and held-out WAWTest (51 MRIs) cohorts - External validation on BraTS-V (125 MRIs) - Open-Source Code on GitHub	On adult glioma validation (BraTS-V) with BraTS model: - DICE 0.896 - H95 5.56 mm - Sensitivity 0.891 - Specificity 0.975 - volumetric ICC 1.000 Additional metrics provided on held-out pediatric OPG and on pediatric OPG with a BraTS model, WAW ensemble, pre-trained ensemble, and transfer-learn ensemble.	- Small, less explored OPG datasets

## AI in treatment decision-making

AI holds growing promise in enhancing precision treatment decisions for patients with NF1-associated glial tumors. One of the most promising current applications of AI in NF1 tumor management is radiomics-based tumor classification. In contrast, the prediction of response to treatment and risk stratification for NF1-associated tumors still requires further development.

Studies have now shown that AI applications distinguish radiomic signatures data with higher accuracy than a radiologist. In a multi-center approach with a larger but mixed cohort including NF1, NF2-SWN, and other SWN patients (*N* = 266), Zhang et al. showed in 2021 that an ML classifier trained with MRI-based radiomics features can better differentiate benign from MPNST than a radiologist does [80]. Ristow et al. further illustrated that benign and MPNST reveal characteristic MRI radiomics features and that an ML classifier can differentiate between those with high sensitivity and specificity [81]. Atypical tumors showed an intermediate feature distribution between benign and MPNST. In another study, Zhang et al. evaluated the utility of an MRI-based ML algorithm for differentiating PNF and schwannomas [80]. However, the interpretation of current NF1 radiomics studies remains subject to some uncertainties, given the relatively small sample sizes. Multicenter studies are needed to investigate the validity and robustness of these signatures in relation to other image acquisition parameters. In clinical routine, morphological criteria such as lobulated appearance, irregular tumor margins, peritumoral edema, and intratumoral heterogeneity are considered worrisome features that indicate a malignant transformation of a PNF into an ANNUBP or MPNST [82]. However, diagnostic accuracy based on morphological features is limited, with reported sensitivities and specificities ranging from 33% to 99% (sensitivity) and 33% to 95% (specificity) [83]. Quantitative imaging, such as DWI, can further enhance diagnostic accuracy; however, despite the advancement of quantitative DWI techniques, including IVIM, there remains a need for improvement.

Thus, radiomic feature-based tumor classification could be used to enhance diagnostic accuracy further and facilitate decision-making for personalized treatments. Several AI-based classifiers achieve high sensitivity and specificity in differentiating tumor subtypes and behavior, and also enable the classification of glioma subtypes and the assessment of survival; however, they lack specificity for NF1 patients to date. Recently, Vershinina et al. [84] presented two explainable ML models for classifying glioma subtypes and predicting survival probability. Although these models only make predictions based on the expression of 13 genes, the results showed high classification and discrimination accuracy. The future will indicate their great potential for personalised treatment strategies and improving prognostic accuracy.

Current rapid developments will bridge AI, multi-omics tumor-based data, and clinical oncology for precise therapy, including NF1-associated tumors  [85]. These quickly developing areas include AI-based improved histotype classification (as demonstrated in extensive international collaborations for gynaecological cancer types  [86], and being in the pipeline for improved glioma subtyping (Ali Bashashati, personal communication), as well as identification of the best drug combination from pre-clincal data, implementing predictions of AlphaFold  [87] that need to be absolutely up-to date for the NF1 protein  [88], and analyzing most expected response to planned therapy  [85]. All these approaches additionally need to be based on standard criteria to achieve the best scientific basis for clinical decision-making.

## Challenges

NF1 presents complex clinical management challenges due to its variable expressivity and multisystem involvement. While AI approaches offer potential solutions for diagnostic accuracy and treatment optimization, several fundamental barriers limit their implementation in NF1.

### Data scarcity

A major challenge is the scarcity of large, well-curated, standardized, and high-quality datasets for training robust AI algorithms. This scarcity manifests at multiple levels: NF1-specific ML studies typically work with cohorts ranging from 39 to 347 patients  [89–91], while systematic reviews of rare disease ML applications show that 35.5% of studies involve fewer than 100 participants  [92]. These sample sizes fall below the minimum threshold of 300 to 1,000 patients typically required to avoid overfitting in medical AI applications  [93, 94].

Geographic disparities in data availability compound the data scarcity problem. The systematic review by Rana et al.  [95] revealed that robust diagnostic data exist primarily in high-income countries, with an absence of relevant research from low- and middle-income countries. This creates a substantial bias in available datasets, limiting the generalizability of AI models to diverse populations and healthcare settings.

The variability in disease manifestation, prevalence, and severity, combined with NF1’s low prevalence, complicates the efficient conduct of clinical trials. Therefore, it restricts the number of study participants and the data to be collected. The European Patient-Centric Clinical Trial Platforms (EU-PEARL) projects specifically identified these challenges as requiring innovative trial designs, including basket-platform trials that can “optimally learn from a small number of potential participants” [96]. The basket-platform trial design proposed for NF1 research acknowledges the need for innovative approaches to overcome data limitations. However, implementing these approaches requires coordination across multiple institutions and countries, presenting logistical and regulatory challenges.

Already existing data accessibility remains problematic. The NF Data Portal  [97], established to address community data-sharing needs, demonstrates both progress and ongoing challenges. By 2019, over 61 public and embargoed NF1, and SWN studies were available, spanning discovery research, translational studies, and clinical data. However, a European landscape analysis by Raycheva et al.  [98] found that only 16.7% of database administrators expressed willingness to contribute data to collaborative research projects. Patient advocacy groups report privacy concerns and unclear downstream use of donated data as major deterrents, underscoring the need for transparent governance and robust anonymization protocols. A possible solution to the privacy concerns could be met by enabling a decentralized model training across multiple institutions without sharing raw data, which is called federated learning. Initial trials in other rare diseases report diagnostic performance of 64% compared with centrally trained models 76%  [99], though system heterogeneity and communication overhead present practical challenges.

Transfer learning provides another possible angle to tackle the data scarcity. Therefore, a DL model is pre-trained on data that is more similar to the target dataset and then fine-tuned on the target dataset, followed by final training with NF1 brain tumor samples. However, the effectiveness of transfer learning is critically dependent on the similarity between source and target domains. For rare diseases like NF1, finding appropriate source domains with sufficient similarity remains challenging. The domain gap between common diseases and rare conditions can greatly limit transfer effectiveness  [100]. Furthermore, while transfer learning aims to address limited data availability, it still requires some annotated target domain data for effective fine-tuning. For NF1, even obtaining this minimal annotated dataset can be challenging, particularly for specialized manifestations like plexiform neurofibromas or optic pathway gliomas. Meta-learning frameworks could help in overcoming this issue. Model-Agnostic Meta-Learning (MAML)  [101] can train models to rapidly adapt to new NF1 subtypes using as few as 5–10 annotated examples, offering a path forward when even small NF1 imaging datasets are unavailable. Another option in overcoming the issue at hand might be in combining imaging, genomic, and clinical data through multimodal DL to compensate for limitations in any single modality. However, this approach is only feasible when multimodal data is available.

Another AI solution to data scarcity is synthetic data generation using Generative Adversarial Networks (GAN) or with additional information provided to a GAN, which can augment the training dataset with corresponding annotations. Recent applications demonstrate success in healthcare contexts, with cGAN (31%) and CycleGAN (18%) being the most utilized architectures  [102]. However, ensuring that generated images accurately represent the diverse characteristics of actual patient data is a major concern, as biases present in training data might also be propagated to synthetic images. Furthermore, there is no consensus on quantitative metrics for validating synthetic NF1 data. Current studies rely on subjective visual Turing tests or downstream classification accuracy, which do not guarantee clinical relevance  [103]. Until standardized evaluation frameworks are widely adopted, synthetic data generation in NF1 will remain an experimental adjunct rather than a reliable solution  [104].

### Ethical and clinical implementation

The use of AI in precision medicine for NF1 raises ethical concerns regarding data privacy, bias, and accountability. AI algorithms require access to large amounts of medical data, underscoring the importance of rigorous data governance, protecting patient privacy, ensuring informed consent for data use, and adhering to legal and institutional data protection standards (e.g., General Data Protection Regulation in the European Union). Furthermore, the data used to train AI algorithms may be biased or incomplete, leading to inaccurate diagnoses or treatments [105]. Given the rarity and heterogeneity of NF1 manifestations, biased models could disproportionately affect patient subgroups, undermining equity in care  [106–108].

As AI systems become more complex and autonomous, questions about accountability and responsibility arise. Who is responsible if an AI system makes a wrong diagnosis or treatment decision? The “black box” nature of many DL models challenges transparency and accountability; in clinical decision-making, explainability is not optional - physicians must understand the rationale behind AI recommendations to trust them, particularly when decisions may influence high-risk treatments such as surgery or MEK inhibition. If an AI-driven recommendation leads to a poor outcome, it is unclear whether responsibility lies with the clinician, the AI developer, or the institution. These concerns necessitate clear regulatory frameworks and robust clinical validation of AI tools before deployment. Bleher et al.  [109] gave three suggestions on how to manage responsibility diffusions: i) provide chances for control, ii) aim to achieve an inclusive and collaborative approach, and iii) facilitate dependable fault management by considering both technical and social dimensions within the realms of complaint, compassion, and recognition  [107–111].

Explainable AI (XAI) provides a partial response to these challenges by enhancing transparency in variant detection, classification, and treatment recommendations. Improved explainability supports clinical accountability, as clinicians can interrogate and justify AI-driven outputs, thereby fulfilling epistemological responsibility and due diligence. XAI can also provide audit trails for institutions and regulators, strengthening trustworthiness in clinical practice and offering mechanisms for post hoc evaluation if adverse outcomes occur.

Informed consent extends beyond data use to the disclosure of AIs role in patient care. Ethical analyses emphasize that patients should be made aware when AI tools contribute to diagnosis or treatment planning, especially when the stakes involve significant risk or when patients retain meaningful agency. In NF1, where AI-guided recommendations may influence surgical decisions or MEK inhibitor therapy, explicit consent may be warranted. However, overly detailed disclosures risk overwhelming patients with technical complexity. A proportionate approach is therefore recommended: robust notification as a minimum standard, with explicit consent reserved for high-risk or high-agency scenarios  [108–110, 112].

Data privacy represents a further challenge in NF1, where genetic and phenotypic information is particularly sensitive. Rare disease datasets heighten the risk of re-identification, and the necessity of multi-institutional data-sharing increases vulnerabilities to data breaches. Moreover, XAI methods that highlight salient features for predictions may inadvertently reveal sensitive or identifiable genotype–phenotype correlations. Safeguarding patient data thus requires not only compliance with existing regulatory frameworks but also the adoption of advanced technical measures such as federated learning, differential privacy, and secure multi-party computation to balance data utility with confidentiality  [107–109].

Addressing these challenges requires interdisciplinary ethical oversight, patient and public engagement, and the integration of ethical-by-design principles into AI development pipelines. To be ethically and practically viable, AI tools for NF1 care must be safe, transparent, fair, and clinically effective.

## Conclusion

Among the discussed applications, radiomics-based classification is the most promising current application of Artificial Intelligence (AI) in the management of Neurofibromatosis Type 1 (NF1) tumors. Molecular genetic AI-based tumor classifiers are already in everyday use, but are not explicitly established for NF1-associated glial tumors. The same is true for AI-based classification from histological slides, which is rapidly growing in pathology; however, data on NF1-associated tumors are currently missing. There is also a gap in validated clinical AI applications for NF1, making this an important area of research for the future.

To date, imaging heterogeneity, variable Magnetic Resonance Imaging (MRI) protocols, and small cohort sizes limit reproducibility and model generalizability. Moreover, ethical concerns such as data privacy and patient consent further complicate the issue of broad data-sharing. Model explainability and clinical trust are additional concerns, as clinicians must understand and validate AI-generated decisions to integrate them confidently into patient care.

Future research should focus on developing multi-center, prospective trials incorporating AI tools into clinical workflows. Such studies should explore the longitudinal predictive value of imaging biomarkers, investigate delta radiomics (tracking feature changes over time), and integrate multi-omics data to support a truly personalized approach. Emphasis should also be placed on Explainable Artificial Intelligence (XAI) systems to ensure clinicians understand and trust the outputs.

We strongly recommend fostering interdisciplinary collaboration between clinicians, data scientists, and AI developers to drive these advancements. Collaborative data platforms (e.g., SYNAPSE, CTF Data Portal) should be leveraged to harmonize datasets and annotation standards. Clinician input is vital for developing clinically relevant features and validating model outputs, while AI experts can ensure technical robustness and scalability. Establishing shared goals, communication protocols, and iterative development cycles will be essential for translational success.

Additionally, developing interpretable AI systems is a challenging task. As more interpretable models may sacrifice accuracy, more complex models may be difficult to interpret. It will require clinicians and computer scientists to work closely to bring XAI to the clinical field and their domain perspective on explanations. Amann et al.  [113] stated that the explanations must be suitable in their respective field of use. Several factors (e.g., time-critical, user knowledge) influence the need for explanations before actions are required.

## Data Availability

Not applicable.

## Abbreviations

18FDG: 18F-fluorodeoxyglucose
AI: Artificial Intelligence
ANNUBP: Atypical Neurofibromatous Neoplasms of Uncertain Biologic Potential
CNS: Central Nervous System
CT: Computed Tomography
DL: Deep Learning
DWI: Diffusion-Weighted Magnetic Resonance Imaging
ERN: European Reference Network
FFPE: Formalin-Fixed Embedded Tissue
GAN: Generative Adversarial Network
GENTURIS: non-Optic Pathway Gliomas
MPNST: Malignant Peripheral Nerve Sheath Tumors
MRI: Magnetic Resonance Imaging
ML: Machine Learning
NF: Neurofibromatosis
NF1: Neurofibromatosis Type 1
OPG: Optic Pathway Gliomas
PET: Positron Emission Tomography
PNF: Plexiform Neurofibroma
PNS: Peripheral Nervous System
RAPNO: Response Assessment in Paediatric Neuro-Oncology
SIOPE: European Society for Paediatric Oncology
SWN: Schwannomatosis
WB: Whole-Body
WHO: World Health Organization
WSI: Whole-Slide Image
XAI: Explainable Artificial Intelligence
